# Rehabilitation medicine summit: building research capacity Executive Summary

**DOI:** 10.1186/1743-0003-3-1

**Published:** 2006-01-03

**Authors:** Walter R Frontera, Marcus J Fuhrer, Alan M Jette, Leighton Chan, Rory A Cooper, Pamela W Duncan, John D Kemp, Kenneth J Ottenbacher, P Hunter Peckham, Elliot J Roth, Denise G Tate

**Affiliations:** 1 Harvard Medical School/Spaulding Rehabilitation Hospital; Boston, MA, USA; 2 National Institutes of Health; Bethesda, MD, USA; 3 Boston University; Boston, MA, USA; 4 University of Washington; Seattle, WA, USA; 5 University of Pittsburgh; Pittsburgh, PA, USA; 6 University of Florida; Gainesville, FL, USA; 7 Powers Pyles Sutter & Verville PC; Washington, DC, USA; 8 University of Texas Medical Branch/Galveston; Galveston, TX, USA; 9 Case Western Reserve University; Cleveland, OH, USA; 10 Rehabilitation Institute of Chicago; Chicago, IL, USA; 11 University of Michigan; Ann Arbor, MI, USA

## Abstract

The general objective of the "Rehabilitation Medicine Summit: Building Research Capacity" was to advance and promote research in medical rehabilitation by making recommendations to expand research capacity. The five elements of research capacity that guided the discussions were: 1) researchers; 2) research culture, environment, and infrastructure; 3) funding; 4) partnerships; and 5) metrics. The 100 participants included representatives of professional organizations, consumer groups, academic departments, researchers, governmental funding agencies, and the private sector. The small group discussions and plenary sessions generated an array of problems, possible solutions, and recommended actions. A post-Summit, multi-organizational initiative is called to pursue the agendas outlined in this report (see Additional File 1).

## 

The advancement of medical science depends on the production, availability, and utilization of new information generated by research. A successful research enterprise depends not only on a carefully designed agenda that responds to clinical and societal needs, but also on the research capacity necessary to perform the work. Research that is likely to enhance clinical practice presupposes the existence of a critical mass of investigators working as teams in supportive environments. Unfortunately, far too little research capacity of that kind exists in rehabilitation medicine to ensure a robust future for the field. The "Rehabilitation Medicine Summit: Building Research Capacity" was conceptualized as a way of fashioning a long-term plan to foster the required developments (see Additional File 1).

## Objectives

The general objective of the summit was to advance and promote research in medical rehabilitation by making recommendations to expand research capacity. More specific objectives were to: 1) bring together leaders in medical rehabilitation research to characterize current research capacity in the field and identify obstacles to expanding that capacity; 2) propose specific actions and mechanisms to enhance research and the development of capacity; 3) formulate an action agenda for use by stakeholders in medical rehabilitation to enhance existing research and training programs or to create new ones; and 4) stimulate federal agencies and foundations to support the needed elements of rehabilitation research and training. Although the purpose of the summit was not to discuss a specific research agenda, the above objectives were considered in the context of five research categories: 1) basic science, 2) clinical research (including clinical trials), 3) outcomes research, 4) health services research, and 5) engineering and technology development.

## Research capacity: operational definition and elements

For the purpose of the discussions, building research capacity was defined as, "a process of individual and institutional development which leads to higher levels of skills and greater ability to perform useful research"[1]. Five elements of research capacity were identified and used to guide the pre-Summit work and the Summit discussions. These included: 1) researchers (their training, mentoring, recruitment, and retention; the value of a career in research and incentives for research); 2) research culture, environment, and infrastructure (academic institutions, the creation and maintenance of core facilities, the role of chairpersons and deans, collaborations, institutional research administration and social culture, and policies governing incentives and job security); 3) funding (sources, advocacy for changing policies, peer-review procedures, funding mechanisms, grantsmanship and fundraising, timing of funding requests, and conflicts of interest); 4) partnerships with other disciplines and disability consumer groups (the purposes of these partnerships; choices of research topics, disciplines, and consumer groups; modes of participation; and potential conflicts of interest when partnering with industry); and 5) the metrics of research capacity (quality and quantity of the pool of available researchers, the productivity of their research, and its impacts).

## Methodology

Several important activities took place before the Summit convened. The Program Committee had extensive discussions about existing research capacity. Key bibliographic references were identified on the topic of building research capacity and made available to all participants. A special article on the history of rehabilitation research was commissioned. Recognized experts were invited to write articles on each of the five elements of research capacity to serve as a basis for discussion during the Summit. These articles were peer-reviewed and five additional experts wrote detailed responses to them. The Research Committee of the American Academy of Physical Medicine and Rehabilitation (AAPM&R) conducted a survey of researchers in the field to identify problems of research capacity and their potential solutions. Several funding agencies submitted reports of their efforts to build research capacity. Finally, participants were given access to a website where all key information was posted, including the articles mentioned above was posted.

The summit consisted of keynote lectures, paper presentations, and small-group working sessions that took place in Washington DC on April 28 and 29, 2005. Invited participants included leaders in the field, senior and junior researchers, department chairs, deans, research directors, professional organizations (12), government agencies (10), disability consumer groups (6), and multiple medical specialties (7). For the group discussions, the participants were divided into 10 small groups, 10 participants per group, making sure that different points of view were represented in each group. Each element of research capacity was discussed independently by two different groups that were charged with identifying problems, solutions, and recommended actions. Their reports were integrated prior to the Summit's final session that was devoted to presenting the reports to the larger group and to discussing additional recommendations. The following sections summarize the groups' conclusions with respect to each of the five elements of research capacity. A more detailed summary of the problems, solutions, and recommended actions identified by the five integrated groups is available from the corresponding author.

## Problem identification

### Researchers

Capacity building requires the development of a pool of well-qualified researchers. To accomplish this task, issues such as training, mentoring, and placing new investigators must be addressed, as do other issues concerning the recruitment and retention of established investigators. The ideal trainee must have a strong commitment to inquiry and the desire and skill to collaborate with others.

Defining the domain of medical rehabilitation research was singled out as being a paramount requirement for expanding research capacity. The field is inclusive by nature because it receives contributions from the physical, biological, psychological, engineering, and social sciences, hence, the difficulty in delineating it. This predicament is reflected in the different conceptual models that are frequently invoked in discussing the field, including the Institute of Medicine's Enabling-Disabling [2] model and the World Health Organization's International Classification of Functioning, Disability and Health [3].

Difficulties in developing, promoting, and retaining greater numbers of skilled rehabilitation researchers were highlighted as well. Far too few programs exist that provide optimal training in medical rehabilitation research. Reasons for the dearth of training opportunities include a lack of training funds from government agencies and private institutions, a paucity of program models for fostering interdisciplinary collaboration, a lack of appropriate mentoring coupled with standardized training curricula for preparing individuals to be competitive as researchers, and inadequate attention to promoting the retention of minorities, women, and individuals with disabilities.

### Research environment, infrastructure, and culture

Research environment, infrastructure, and culture represent a matrix of complex factors essential for excellence in generating medical rehabilitation research, training, recruiting researchers, and in conducting research involving people with disabilities.

A major problem is the lack of recognition of research and scientific discovery as an institutional, organizational, and professional core value. In too many instances, scientific discovery is not an explicit priority in the vision and mission statements of clinical and professional organizations with national memberships. Consequently, the strategic plans of these organizations do not promote collaborative or interdisciplinary research, and they are not expressly supportive of the necessary investments in scientific training, the development of grant writing skills, and the mentoring of promising research faculty. The human and physical resources to accomplish these tasks are unavailable in many academic rehabilitation environments. Mechanisms to recognize research productivity in formal and informal evaluation and reward systems are frequently lacking as well.

### Funding

Significant funding must be specifically assigned to building research capacity. However, the current economic environment is likely to result in flat or even reduced funding for medical rehabilitation research, at least in the near future. This unfortunate financial picture exists at a time of increasing need associated with the growing number of individuals with disabilities, and of unparalleled opportunities to improve their lives by means of new knowledge generated by research.

The biggest problem is lack of a coherent strategy for advocating the needed research support. Stakeholders in medical rehabilitation research are fractionated in their efforts to obtain larger expenditures. The austerity of the current funding environment underscores the importance of organizations bringing their advocacy efforts together under common goals.

The problem of generating adequate funding for medical rehabilitation research exists at three levels. At the federal level, the field lacks visibility as being a worthy object of support when strategic funding decisions are made. At the local level, only a handful of academic programs have the research infrastructure required to produce successful research, and very few new programs have been developed in the past decade. This partially reflects the fact that many academic medical centers invest most of their resources in expanding the ability of their extant programs to generate research funds, rather than in developing promising new programs such as ones in medical rehabilitation. Finally, at the level of individual researchers, proposed research too frequently lacks the quality to merit being funded. Additionally, some researchers fail to take advantage of existing opportunities for funding, simply because they do not know of their existence.

### Partnerships

Partnerships with scientists in other disciplines, academic departments, and institutions, and with consumers with disabilities, among others, are vital to enhancing the capacity for conducting high quality, meaningful research. Several factors have limited the development of those partnerships. Because of the diversity of stakeholders and stakeholder objectives, a common framework has been lacking upon which to build funding, policy, programmatic, and marketing messages regarding research. Nor have consistent efforts been made to ensure the meaningful participation of individuals with disabilities in the research process.

### Metrics

Concerted efforts to enlarge the capacity of medical rehabilitation research must be complemented by an ability to assess that capacity over time in order to gauge progress. No constitutive definition of research capacity appears to have won broad endorsement in the health sciences literature, and little guidance exists for deciding on the metrics and measures for its principal domains. Notwithstanding the lack of precedence, the meaning of medical rehabilitation research capacity must be understood with precision if that capacity is to be rigorously and comprehensive assessed.

## Solutions and recommended actions

Although each group worked independently on its assigned problems, many of the solutions and recommended actions they identified were quite similar. This section integrates the solutions and recommended actions.

### Coalition

Several discussion groups suggested the formation of a coalition of professional groups and consumer organizations. This coalition would create a national agenda addressing the issues of funding, capacity-building needs, and public education and awareness. It would develop specific objectives and action plans regarding 1) funding targets for research and research training, 2) needed changes in funding agencies' policies and practices, and 3) initiatives to educate the public about the importance and societal benefits of rehabilitation research, and it would coordinate efforts to address those issues.

### Training

A high priority area is the training of new investigators. To accomplish this goal, training curricula need to be created, and funding needs to be expanded for rehabilitation research training programs across disciplines and at multiple levels, including undergraduate students, students in professional training program, faculty, and department chairs. Special efforts should be made to recruit and train women, students with disabilities, and minorities.

### Career paths

Researchers need support at different stages in their careers. Current funding sources fail to provide the needed continuity of support as their careers evolve. To foster researchers' development and their retention in the field, funding opportunities must be increased for pre-doctoral students, post-doctoral fellows, junior faculty, and established faculty transitioning into new investigative areas.

### Partnerships to conduct research

To assure its scientific importance and clinical relevance, rehabilitation research requires both interdisciplinary and multi-stakeholder partnerships. Collaborations among researchers of different scientific and professional disciplines need to be promoted and cultivated. The required initiatives must come from individual researchers as well as from professional organizations that encourage joint scientific meetings and discussions of interdisciplinary research issues. Partnerships are vital, too, to assure that rehabilitation research is informed by the perspectives of its intended beneficiaries – individuals with disabilities, their family members, and rehabilitation practitioners. Principal investigators should implement Participatory Action Research (PAR), making it an integral part of medical rehabilitation and disability research. Greater emphasis should be placed as well on providing people with disabilities with the training and support necessary for them to assume leadership roles in rehabilitation research.

### Infrastructure

Currently, only a handful of departments or centers have the research personnel, equipment, space, and support staff that constitute a strong infrastructure for medical rehabilitation research. Many more such programs must be established before the aggregate research capacity is commensurate with existing knowledge needs. Inevitably, that will require host institutions to invest in establishing new rehabilitation research programs or in strengthening ongoing ones. A growth strategy should be pursued concurrently of building intra-institutional partnerships that facilitate access to the infrastructure available to colleagues in other scientific and professional disciplines.

### Message to funding agencies

Funding agencies do not assign sufficiently high priority to medical rehabilitation research. Within the NIH, this can be rectified by establishing an independent institute dedicated to rehabilitation research. Actions are needed as well to expand the participation of rehabilitation scientists in scientific review panels, and to generate more requests for applications that focus on interdisciplinary rehabilitation research. A farther-reaching possibility is creation of an independent agency for disability issues within the Department of Health and Human Services. Advocacy directed at federal agencies must be complemented by initiatives aimed at increasing support from private-sector sources such as third party payers.

### Rehabilitation science model

It is generally accepted that the field lacks a unified scientific model. A consortium of experienced researchers should be created to develop this model and to define the domains and boundaries of rehabilitation research.

### Mission statements and strategic plans

Scientific discovery is not always recognized as an institutional or organizational core value. Professional organizations should include research as an important component of their mission statements. This should be reflected in their strategic plans and used as a means to promote interdisciplinary and collaborative research.

## Metrics

Both long-term and short-term perspectives are called for to meet the challenges of assessing medical rehabilitation research capacity. The long-term perspective highlights the definitional and operational challenges that must be addressed eventually if that capacity is to be rigorously conceptualized and comprehensively assessed. The short-term outlook emphasizes that some information gathering can and should begin immediately in the following four areas.

1. *Rehabilitation Research Trainees*. Information to be tracked includes: the number of funded post-doctoral positions available in rehabilitation and the distribution of fellows across rehabilitation disciplines; the proportion of trainees who come through research training programs and who become researchers – full, part-time, or none; the research products that the trainees generate, as well as their extramural and intramural levels of funding. Possible action steps include defining who is considered as a core rehabilitation professional, exploring and using where possible existing methodology, and enlisting the cooperation of funding agencies to collect and share this information.

2. *Size of the Rehabilitation Research Cadre*. Information to be tracked includes the size of academic departments relevant to medical rehabilitation (e.g., number of research fellows, filled and unfilled faculty positions), and the amount of time rehabilitation professionals, broadly defined, spend in research, e.g., half-time or more, part-time, or none). Professional organizations should be enlisted to collect this information on a regular and standardized basis.

3. *Productivity*. The information to be monitored includes citations of published articles, extramural and intramural levels of research funding, and the types of research designs appearing in the rehabilitation literature. Action steps include specifying the kinds of articles and the journals to include, and searching by professional organization memberships, institutions, or by disciplines or countries. Professional organizations should be enlisted to collect this information on a regular and standardized basis, using existing methodology where possible.

4. *Federal Agency Expenditures on Rehabilitation Research*. Expenditures allocated to rehabilitation research in specific content areas should be monitored. A recommended action step is to identify agency contact points to secure these data on an annual basis.

The longer-term challenge is to develop a consensually acceptable definition of medical rehabilitation capacity, and then to operationalize each of its key components. Domains that are likely to be encompassed in that definition include *funding, qualified researchers, institutions, research training, research methods, an applicable knowledge base, an encompassing research agenda (including topics, their relative priority, and funding levels), knowledge translation activities, defined consumer demand and need, and political advocacy*. The figure is an attempt to organize those domains within a coherent framework. Each domain is assigned to one of three categories – the Research Agenda, Research Environment, or Researchers – or to the conjunction of two of these groups. Steps should be taken to refine that schematization along with the separate domains comprising it. Additionally, feasible means must be identified to 1) quantify each domain and 2) characterize its quality of achievement (against some standard or norm). It will be necessary then to establish the psychometric properties of the key indicators, e.g., their validity, reliability, and sensitivity.

A post-Summit, multi-organizational initiative is called for to pursue the agendas outlined above. Data-gathering efforts should be launched as soon as possible to characterize current research capacity as a baseline for assessing possible future gains. Those efforts should draw on findings of the Survey on Academic Leadership and Research Development conducted by the Research Advisory & Advocacy Committee of the AAPM&R, and be implemented by either 1) an ensemble of federal agencies supporting rehabilitation research, or 2) a consortium of rehabilitation-related voluntary organizations such as those represented at the Summit.

**Figure 1:**
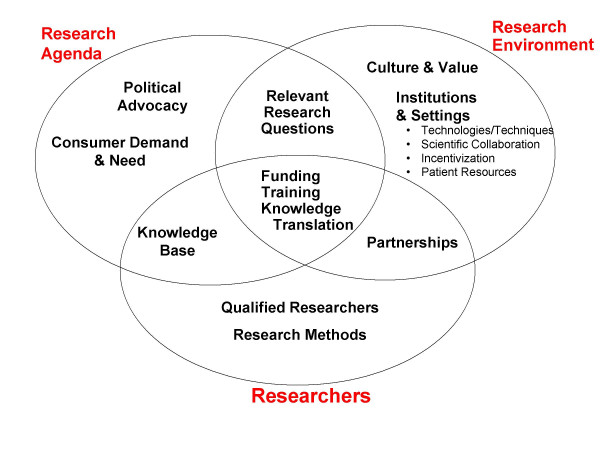
A taxonomy of research capacity as a guide for knowing what to measure.

## Editor's note

This article will be published almost simultaneously in the following journals: American Journal of Occupational Therapy; American Journal of Physical Medicine and Rehabilitation; American Journal of Speech Language Pathology, Archives of Physical Medicine and Rehabilitation; Assistive Technology; Burn Care and Rehabilitation; Disability and Rehabilitation; Journal of Musculoskeletal Pain; Journal of NeuroEngineering and Rehabilitation (online); Journal of Rehabilitation Research and Development; Journal of Spinal Cord Medicine; Neurorehabilitation and Neural Repair; OTJR: Occupation, Participation, and Health; Physical Therapy; The Journal of Head Trauma and Rehabilitation; Topics in Stroke Rehabilitation

## Supplementary Material

Additional File 1 A table outlining the Final Action Plan of the Rehabilitation Medicine Summit: Building Research Capacity held on April 28–29, 2005 in Washington, DC. Click here for file
