# Supplementary material for: Rehabilitation medicine summit: building research capacity Executive Summary
Source: J Neuroeng Rehabil. 2006 Jan 3;3:1. doi: 10.1186/1743-0003-3-1 (PMC1351393; doi:10.1186/1743-0003-3-1)
Supplement: Additional File 1 — A table outlining the Final Action Plan of the Rehabilitation Medicine Summit: Building Research Capacity held on April 28–29, 2005 in Washington, DC. [file 1743-0003-3-1-S1.doc]

| Rehabilitation Medicine Summit: Building Research Capacity April 28-29, 2005, Washington DC Final Action Plan | | | |
| --- | --- | --- | --- |
| Research Capacity Element | Researchers | | |
| Group Leaders | Hunter Peckham, PhD and Denise G. Tate, PhD | | |
|  | | | |
| Problem | | Solution | Recommended Action |
| 1. **Lack of definition of the domains of rehabilitation science.** | | 1. Develop/evaluate proposed models of rehabilitation science (multidisciplinary, IOM, others). | 1. Convene a group of “conceptualizers” with inclusion from various stakeholders to examine different models and relate the outcomes to research training models.  2. Develop a white paper or report that lists potential successful models of rehabilitation science based on the outcomes of this meeting.  3. Participate in a rehabilitation consortium that would address the definition of rehabilitation science, including research training issues and implications for the field. |
| 2. Lack of exposure to rehabilitation and rehabilitation research and need to create a research environment aligning recruitment practices to address **insufficient number and quality of researchers.** | | 1. Short-term undergraduate and graduate funding options such as summer programs for exposing non-rehabilitation researchers to rehabilitation research and rehabilitation scientists to new research trends.  2. Extend research training duration under current training programs available by our current funding agencies. | 1. Ask organizations to problem solve how to more effectively highlight research through plenary sessions and invitations to cutting edge speakers.  2. Encourage joint meetings on common scientific themes.  3. Foster relationships with Schools of Public Health to expand training opportunities.  4. Develop a “suite” of mechanisms for pre-candidates with potential to be trained in rehabilitation research to incorporate clinicians into research activities. |
| 3. **Lack of training funds** | | 1. Expand financial support for research training. | 1. Advocate for funds for research training – this might be best achieved through a joint coalition with especial attention to career development avenues.  2. Expand the rehabilitation research networks approach for specific research training in specific scientific domains including specific laboratories and research environments  3. Explore private donor relationships to support trainees’ stipends. |
| 4. Lack of available program **models fostering interdisciplinary** **collaboration** | | 1. Create venues for interdisciplinary collaborations across departments and universities. | 1. Require that existing training grants (such as the NIH T32 and K12’s) include a multidisciplinary training focus for research. |
| 5. **Lack of appropriate mentoring** | | 1. Create and develop guidance materials.  2. Teach mentees how to select and use mentors. | 1. Emphasize the scope of mentorship and develop guidance materials (career advisement, scientific and professional).  2. Identify models of mentoring from other specialties and organizations ( Psychiatry, Geriatrics, Pediatrics, National Science Foundation Advance programs) |
| 6. Lack of a standard **scientific training curriculum** | | 1. Develop standard protocols for research and a related curriculum for training in rehabilitation research. | 1. Review current research training curricula and ensure that it includes clinical trials training.  2. Utilize the consortium mechanism to review curriculum approaches to ensure the scientific mission of our disciplines |
| 7. Lack of strategies for **retention** of vulnerable groups (women, minorities, consumers) | | 1. Gain a greater awareness of problems that impede retention (i.e. rules for promotion). | 1. Establish a multidisciplinary task force to review these problems. |
| 8. Preparation of individual for her/his **role** (how to get researchers to be competitive?) | | 1. Develop an adequate career path for retention of rehabilitation scientists | 1. Establish special interests groups (SIG’s) related to developing materials and communicating strategies.  2. Develop joint appointments that promote career development. |
| 9. Lack of **critical mass** of researchers | | 1. Develop strategies for bringing together a critical mass of researchers in our field. | 1. Support efforts to modify the way agencies list departmental and institutional funding so it will create incentives for collaborative research. |

| Research Capacity Element | Research Environment, Infrastructure, and Culture | | |
| --- | --- | --- | --- |
| Group Leaders | Kenneth Ottenbacher, PhD and Elliot Roth, MD | | |
|  | | | |
| Problem | | Solution | Recommended Action |
| 1. Lack of explicit **prioritization of research in mission statements.** | | 1. Incorporate research into mission statements of rehabilitation entities and revisit relative position of research as a component of the mission. | 1. National organizations to re-visit the position of research in their mission statements.  2. National organizations to develop sample mission statements for use by organizations and institutions. |
| 2. Lack of **strategic planning** that supports collaborative and interdisciplinary research and is consistent with the resources and institutional culture | | 1. Develop strategic plans for targeted research themes. | 1. Professional organizations and individual institutions to develop strategic plans for targeted research areas and building coalitions.  2. Identify and disseminate examples of successful strategic plans |
| 3. **Beliefs, attitudes, and values** that do not support research | | 1. Establish cross-discipline successful models. | 1. Identify, utilize and disseminate database of “research evangelists.”  2. Develop database of successful researcher-clinician role models and successful research institutions.  3. Publicly recognize research success in organizations.  4 Develop cross-discipline articles and workshops on the topic of research culture to include in specialty journals and conferences. |
| 4. Inadequate **incentives and rewards** for research. | | 1. Develop incentive and reward programs that incorporate research | 1. Develop and disseminate models for providing monetary and non-monetary incentives.  2. Recognize institutions and organizations that have adopted successful models.  3. Establish research incentive consultation teams. |
| 5. Lack of scientific **training**, grantsmanship, and senior faculty to serve as **role models**. | | 1. Increase training opportunities and number of senior investigators with successful research programs. | 1. Increase career and faculty development programs (such as the NIH’s K12, K30, K02, K05, and K07 type awards). Develop partnerships with other entities to fund such programs. Encourage development and expansion of fellowship programs for senior faculty (such as NIDRR’s).  2. Encourage institutions to recognize mentoring as a faculty responsibility; provide credit and incentives in faculty evaluation process for successful mentoring.  3. Create task force to identity needs and existing research courses and workshops in the field. Identify most successful courses and sponsors. List courses and workshops on a web site. Develop list of faculty willing to serve as mentors with their area of expertise.  4. Develop funding (internal and external) to provide extended research experiences in high priority (hot topic) areas for students, residents and fellows or for senior faculty who want to re-tool. |
| 6. Lack of **infrastructure** that supports collaborative and interdisciplinary research and is consistent with the resources and **institutional culture.** | | 1. Develop strategic plans to enhance local and national infrastructure. | 1. Professional organizations and/or foundations to establish a consultation model/service to help new or small departments develop research programs. PM&R Foundation to consider subsidizing consultation program.  2. Identify, develop and disseminate a database of successful models of organizational infrastructure including resources such as “toolkit” and web-based resources. |
| 7. Chairs and faculty leaders often lack **experience and skills in developing and maintaining successful research programs**. | | 1. Create and/or expand training programs for faculty leaders | 1. Professional organizations and foundations in rehabilitation should expand existing training programs for chairs and faculty to include more emphasis on research.  2. Identify and publicize existing training programs for chairs offered by research intensive universities (e.g. program at Harvard) and organizations (e.g. AAMC).  3. PM&R Foundation develop program to subsidize faculty chairs attending leadership training programs.  4. Identify and disseminate existing and potential models of leadership that promote rehabilitation research.  5. Establish mentorship and coaching models to develop research leaders.  6. Create a research development consultation team. |
| 8. Lack of **visibility and identity** limits opportunities for collaboration with potential academic and industry partners. | | 1. Enhance visibility and recognition in targeted arenas: academic, general public, industry, etc. | 1. Expand existing marketing plans and efforts to highlight research as well as clinical contributions to society.  2. Invest in development of public relations program using professional consultants focused on current and potential future contributions of rehabilitation researchers in specific hot topic targeted areas. |

| Research Capacity Element | Funding | | |
| --- | --- | --- | --- |
| Group Leaders | Leighton Chan, MD, MPH and Pam Duncan, PhD | | |
|  | | | |
| Problem | | Solution | Recommended Action |
| 1. Lack of **advocacy** for research support | | 1. Mobilize population(s) to advocate for rehabilitation research including people with disabilities. | 1. Organize a summit for constituency organizations (for example, NMSS, AARP, and others) to join efforts and support rehabilitation research.  2. Form “Friends of Rehabilitation Research”  campaign to highlight voters with disabilities living in your community  3. Emphasized demographics of disability |
| 2. Lack of **rehabilitation penetration** in federal issues | | 1. Implement scientific review panels with expertise and interest in rehabilitation.  2. Develop more research centers of excellence.  3. Become more influential and engage NIH networks.  4. Consider a non-NIH Federal Agency to consolidate federal disability organizations in the Department of Health and Human Services (including more support for rehabilitation research) | 1. Request NIH dedicated rehabilitation permanent scientific review panel.  2. Issue a request for applications (RFA) for additional “Interdisciplinary Research Centers of Excellence.”  3. Organize meetings of leaders of rehabilitation organizations (a coalition) with the Directors of NIH and other funding agencies.  4. Ask NIH to send out RFAs related to rehabilitation research across Institutes as a development tool for capacity of less experienced researchers.  5. Given disparity in Federal funding agencies for disability, consider legal opinion to pursue Federal Government for Discrimination against People with Disabilities.  6. Develop a group of accomplished researchers (Speaker’s Bureau) who would be willing to speak to funding agencies as needed to discuss research funding, training, and overriding issues  7. Move beyond NIH to develop a Disability Agency in DHHS (incorporates medical, social, transportation, and other issues) |
| 3**. Fragmentation** of rehabilitation organizations and **lack of coordination** among organizations (PM&R, PT, OT, Neuroscience) | | 1. Consolidation of rehabilitation organizations to create a focused voice on rehabilitation research. | 1. Get the AAPM&R, AAP, and ACRM to develop a plan to coordinate the efforts of rehabilitation organizations.  2. Create a united voice with participation of all organizations (PM&R, Neuroscience, Allied Health Professions) |
| 4. Lack of departmental **resources for infrastructure in local institutions and medical schools.** Recognition of financial resources rather than science. | | 1. Center Grants for institutional infrastructure supporting rehabilitation research are needed.  2. Obtain the support of the leadership including Deans of professional schools. | 1. Meet with AAMC representatives and leadership to advocate for encouragement, visibility, and funding in Medical Schools for rehabilitation research infrastructure.  2. Consider new branding (hire professional consultants) of rehabilitation efforts to be more visible, consistent, inclusive, and emphasize research. |
| 5. Quality of research and **competitiveness** of individual researcher (Do we walk the walk to get funding?) | | 1. Local institution must value individual researcher to be competitive  2. Make resources available to develop quality grants | 1. Look within ourselves and accept responsibility to be more competitive researchers and seek collaboration across disciplines.  2. Teach the art of networking outside of our own department and grantsmanship.  3. Conduct mock study section reviews “in house” to refine grants. |
| 6 We can **better identify other funding sources**. | | 1. Identify other funding sources | 1. Foundation for PM&R, AAP, AAPM&R, ACRM, and other professional organizations to develop web page listing of all possible resources (e.g.—Paralyzed Veterans of America, National Stroke Association, Foundations, and others)  2. Develop case studies of rehabilitation researchers who have been successful in obtaining funding.  3. To develop a portfolio of funding options. |

| Research Capacity Element | Partnerships | | |
| --- | --- | --- | --- |
| Group Leaders | Rory Cooper, PhD and John Kemp, JD | | |
|  | | | |
| Problem | | Solution | Recommended Action |
| 1. **There are diverse stakeholders and a lack of a common framework.**   Lack of **unified research vision** among rehabilitation research partners. There needs to be **a national agenda** from “the field” on disability and rehabilitation policy. How does length of stay and rehabilitation outcomes effect the cost to the family, community, and society? | | 1. Try to bring together the Federal Agencies, State Agencies, Professional Societies, Consumer Organizations, Foundations, and Research Institutions. Develop effective partnerships with and among these organizations.  2. Try to bring together the diverse professional societies to agree upon key issues and strategies for areas of common interest. For example, those professional societies participating in this summit.  3. Form partnerships with different Departments and Professions. Incorporate industry as part of the partnership where sensible. Work with state organizations.  4. Much larger group needs to demand funding. Only through partnering with consumers (disability organizations, individuals with disabilities, advocacy organizations) can there be a large and effective enough group to increase funding. Organizations of people with disabilities need to be brought together.  5. Educate foundations and other organizations about disability and medical rehabilitation research.  6. We need to form partnerships to educate the public and public officials to remove social stigma of disability and to understand the value of research. | 1. Form a “Rehabilitation Coalition” to speak with a common voice. This would likely have to be issue or project focused. For example, to promote research and capacity building. The American Institute of Medical and Biological Engineering (AIMBE) or ITEM Coalition may be models to consider.  2. Create Educational Programs to inform the various stakeholders: professional organizations and consumers, and to seek their support for rehabilitation research and research policy.  3. Create educational programs for consumers and non-rehabilitation professional groups about the benefits and positive outcomes of rehabilitation research. For example, increased risk of cardiovascular disease among people with SCI.  4. Establish an annual forum.  5. Establish a national partnership body, comprised of:   - Government   - ICDR   - Research regulation and reimbursement - For-profit sector   - Venture capital   - Pharma   - Insurance - Voluntary consumer organizations   - Health   - Rehabilitation - Professional organizations - Legislative organizations - Academic community |
| 2. **Inadequate full participation of consumers in research and development.** | | 1. Include “Relevance to the Consumer Population” and “Significance to the Consumer” as part of the grant process in every funding agency.  2. Include educated consumers in the peer-review process.  3. Provide incentives to investigators to include people with disabilities.  4. Greater communication with consumers needs to be established. We need to institutionalize consumer driven research priorities, and we need to regularly and effectively communicate R&D results to consumers.  5. Partnering with groups that can provide necessary funds.  6. Education of general public about the potential to ameliorate or live with a disability | 1. Develop training programs for people with disabilities (including family members) in order to promote meaningful participation in rehabilitation research.  2. Expand scholarship opportunities within federal agencies and private foundations for people with disabilities.  3. Create an awareness campaign so that consumers become knowledgeable about opportunities to contribute to research and development.  4. Partner with consumer groups (AAPD, NCIL, etc.) and other health advocacy groups  5. Conduct research in public policy  6. Seek dedicated funding to enhance partnerships  7. Support a disability leadership summit on research  8. Train researchers and proposal writers how to tap into priorities and consumer expertise.  9. Encourage PI’s to implement participatory action research. |
| **3. Lack of relationships with payers results in discrimination towards people with disabilities.** | | 1. Partnerships with reimbursement organizations (for example to show that the 75% rule is flawed there are cost implications and community participation issues. This dialogue needs to be inclusive of more groups of people with disabilities).  2. Dialog with CMS and other health care providers about provisions of specific services for people with specific disabilities (in the home rule). | 1. Have discussions with payers prior to the research projects.  2. Request the IoM to look into the 75 percent rule and the “In the Home Rule” again to prevent people from being shuffled-off to Nursing Homes.  3. Promote a “call for research” to determine the best “rehabilitation processes and structures” and the possible impacts of proposed policy changes. For example, what are the best combinations of rehabilitation services and settings to achieve optimal (acceptable) outcomes for people with various impairments. |
| **4. Lack of rehabilitation research capacity and rigor.** | | 1. Partnering with other relevant disciplines (medical specialties, allied health professionals).  2. Funded networks with adequate resources to conduct collaborative, rigorous research.  3. Partnership with patients to create cohorts for long-term follow-up.  4. Increasing rehabilitation exposure to medical students | 1. Leadership training for department chairs.  2. Partner with institution to develop an infrastructure for rehabilitation research.  3. Conduct research in the mechanisms by which treatments work – basic science.  4. Special incentives for new investigators.  5. Partnership with patients to create cohorts for long-term follow-up.  6. Mentoring, developing a presentation to give to medical students to encourage rehabilitation research |

| Research Capacity Element | Metrics |
| --- | --- |
| Group Leaders | Marcus Fuhrer, PhD and Alan Jette, PhD |

| Problem | Solution | Recommended Action |
| --- | --- | --- |
| 1.Lack of a **consensual definition** of “research capacity” | 1. Define the construct by delineating its essential components, some attractive candidates being *funding, qualified researchers, institutions, research training, research methods, an applicable knowledge base, an encompassing research agenda (including topics, their relative priority, and funding levels), knowledge translation activities, defined consumer demand and need, and political advocacy.* | 1. Submit the array of domains to an intensive review to assure that is reasonably comprehensive and free of redundancies. |
| 2. Lack of **conventions for deciding on metrics and measures for many of the domains.** | 1. Attain consensus on feasible ways to a) quantify each domain and b) characterize each domain’s quality of achievement. Then establish the psychometric properties of the key indicators, e.g., their validity, reliability, and sensitivity. | 1. Devote the post-Summit, multi-organizational strategic planning effort in part to deciding how to implement the necessary empirical work, both the psychometric development of indicators and their application in a data gathering effort to characterize baseline research capacity. |
| 3.Lack of a database describing **current research capacity as a baseline** for assessing future gains. | 1. Create a database describing current research capacity as a baseline for assessing future gains. | 1. Develop the database, drawing on the AAPM&R/RAAC Survey on Academic Leadership & Research Development, on behalf of either 1) a possible effort to coordinate federal-agencies supporting rehabilitation research, or 2) a consortium of rehabilitation-related voluntary organizations such as those represented at the summit. |
| 4. There may be **too many specific domains**, making it potentially unrealistic to capture them all in a database. | 1. Organize the domains by identifying a subset of underlying ones. | 1. We developed a Venn diagram comprised of three “super-domains” that relate directly to the four other focus-group topics (cf. PowerPoint slide). |

| Indicators of Research Capacity Building |  |  |
| --- | --- | --- |
| **1.Rehabilitation Research Trainees.** | 1. Number of funded post doc positions available in rehab (NIH, NIDRR, VA, CDC, AHRQ and other national funding agencies) and the distribution of fellows across rehabilitation disciplines.  2. Proportion of trainees who come through research training programs who become researchers: full, part time, none.  3. Average research products by research trainees in rehabilitation including citation of research products and extramural & intramural levels of funding | 1. Define who is considered as a core rehabilitation professional.  2. Explore and use where possible existing methodology.  3. Enlist cooperation of funding agencies to collect and share this information |
| 2.**Size of rehabilitation research cadre** | 1. Track size of academic departments of PM&R and beyond: number of fellows, openings, number filled.  2. Measure amount of time rehabilitation professionals, broadly defined, spend in research: 50%+; part time; none. | 1. Enlist professional organizations to collect this information on a regular and standardized basis  2. Secure data from the AAP Annual Survey |
| 3.**Productivity** | 1. Measuring publications by rehabilitation professionals, broadly defined, and citations of published articles; extramural & intramural levels of research funding  2. Track levels of research designs published in rehabilitation literature | 1. Define the articles and journals relevant to include.  2. Could search by professional organization membership, institutions, discipline, and by country.  3. Enlist professional organizations to collect this information on a regular and standardized basis.  4. Explore and use where possible existing methodology. |
| 4.**Funding** | 1. Track federal agency expenditures on rehabilitation research by specific content areas. | 1. Enlist friends of rehabilitation to identify agency contact points to secure these data on an annual basis. |
|  |  |  |

*Note:* IOM = Institute of Medicine, NIH = National Institutes of Health; NIDRR = National Institute on Disability and Rehabilitation Research; PM&R = Physical Medicine and Rehabilitation; AAMC = Association of American Medical Colleges, NMSS = National Multiple Sclerosis Society, AARP = American Association of Retired Persons; ACRM = American Congress of Rehabilitation Medicine, ICDR = Interagency Committee on Disability Research, AAPD = American Association of People with Disabilities, NCIL=National Council on Independent Living, DHHS = Department of Health and Human Services; PI’s = Principal Investigators, CMS = Centers for Medicare and Medicaid Services, VA = Veterans Administration, CDC = Centers for Disease Control and Prevention; AHRQ = Agency for Health Care Research and Quality.
